# What people know about congenital CMV: an analysis of a large heterogeneous population through a web-based survey

**DOI:** 10.1186/s12879-016-1861-z

**Published:** 2016-09-26

**Authors:** Sandro Binda, Laura Pellegrinelli, Marco Terraneo, Alessandra Caserini, Valeria Primache, Laura Bubba, Maria Barbi

**Affiliations:** 1 Department of Biomedical Sciences for Health, University of Milan, Via C. Pascal 36-20133, Milan, Italy; 2 Department of Sociology and Social Research, University of Milano-Bicocca, Milan, Italy; 3 Laboratory of Opinion Polls (LID), University of Milan, Milan, Italy

**Keywords:** Cytomegalovirus, Awareness of congenital cytomegalovirus, Web survey

## Abstract

**Background:**

Congenital CMV (cCMV) infection is a serious public health issue due to both its worldwide prevalence and the severe and permanent impairments it causes. However, awareness of this infection is low in the general population and among pregnant women, and it also seems to be generally disregarded by healthcare providers. The identification of factors behind this inadequate level of knowledge could provide a basis for future preventive measures. This study aimed at evaluating awareness of CMV and cCMV infection and its correlation with socio-demographic variables in a general population.

**Methods:**

The survey was carried out by computer-assisted web interviewing (CAWI). A questionnaire was sent via e-mail to the 70,975 individuals who comprised the whole population (students, administrative staff, teaching staff) of Milan University, Italy in 2015.

**Results:**

Out of the 10,190 respondents, 5,351 (52.5 %) had already heard of CMV but only 3,216 (31.8 %) knew that this virus could be implicated in congenital infection. Urine and breastfeeding were the least recognized transmission routes for CMV infection; less than half of respondents accurately identified the right symptoms and sequelae caused by cCMV infection. The correct hygienic measures against cCMV infection were identified in percentages ranging from 55.6 to 75 % depending on the measures proposed but about one in three of interviewees deemed those measures unnecessary in the event of a pregnant woman already being CMV seropositive. From the mean knowledge scores the most complete quality of awareness of CMV turned out to be linked to childbearing-age (25–40 year) and with not having children, even if results for non-parents showed less of them having heard of cCMV than parents.

**Conclusion:**

Our results indicate a limited and confused awareness of cCMV infection in a large, fairly young and well-educated Italian population.

## Background

Human cytomegalovirus (CMV) is widespread, belongs to the family of herpetic viruses and behaves as an opportunistic pathogen, targeting immunocompromised subjects or those possessing an immature immune system. The infection may be contracted in intrauterine life from the mother, making CMV an important agent of congenital infection. It is estimated that CMV strikes about 0.7 % of live born babies in developed nations [1] and up to 6 % of newborns in developing countries [2, 3]. In about 90 % of infections, symptoms at birth are not shown but 20 % of children with congenital infection either present at birth or later develop permanent damage, in particular, sensorineural hearing loss and psycho-motor retardation [1, 4]. Since no tested vaccines against CMV are licensed as yet, education and counselling about hygienic practices (such as hand washing and avoiding contact with bodily fluids, in particular with children’s urine and saliva) remain the only means of reducing the rate of congenital CMV infection (cCMV) and therefore their implementation is fundamental [5]; however, awareness concerning cCMV seems to be limited to population clusters, even pregnant women and physicians have shown little awareness or knowledge of cCMV [6–11], there is currently only limited information available about the general population [12].

In order to investigate awareness of cCMV infection and its eventual correlation with socio-demographic variables in a general population, we carried out a survey conducted by computer-assisted web interviewing (CAWI) which was sent via e-mail to the whole population attending the University of Milan, Italy. This population was chosen for its size and its considerable heterogeneity in relation to age, gender, level of education and parenthood.

## Methods

### Study population

A brief, written and self-administered web-questionnaire was sent to 70,795 people (students and employees) attending the University of Milan in 2015. The study was carried out by the Virological Diagnosis Laboratory, Department of Biomedical Sciences for Health at the University of Milan, Italy. It was conducted according to institutional university review board regulations and ethical approval was granted.

### Questionnaire development

The computer-assisted web interviewing (CAWI) was designed by the authors as a questionnaire and conducted by the Laboratory of Opinion Polls LID, University of Milan (http://www.socpol.unimi.it/lid) using IdMonitor V 4.9.2 (IdWeb s.r.l, Città di Castello, Italy). The questionnaire was based on those used by Jeon [11] and Cannon [12] which had previously been validated by the expert review with regards to the questions pertaining to CMV awareness. The 7-min questionnaire was anonymous and composed of multiple-choice items and open questions so as to collect information as shown in Table 1. True and false answers were available for multiple-choice items (Table 1). Questions regarding age, level of education, status in the University (student, academic and non-academic staff), gender, and parenthood were also included. Age groups were divided as follows: individuals up to 24, from 25 to 40 and over 40 years. Education was categorized as undergraduate, bachelor’s degree holder and master’s degree holder according to the highest educational level or qualification achieved. Finally, we distinguished between parents (parents and parents-to-be) and non-parents. The enrolment to the study was done by e-mail invitation sent to everyone attending the University of Milan with an easy-to-use link to connect the participants with the questionnaire and thereby acknowledging their agreement to participate in the survey. An announcement about the survey was posted on the ‘homepage’ of the University’s website and on social networks in order to promote the study some days before the publication of the questionnaire. Participants were blinded to the questionnaire topic so as to prevent them from researching CMV and thus biasing their responses.

**Table 1:** Summary of the questionnaire including the possible multiple-choice answers

#1) Have you ever heard about the following conditions?		
#2) Among them, which can be acquired from the mother during pregnancy?		
Fetal alcohol syndrome	Toxoplasmosis	Down Syndrome
Cytomegalovirus	Rubella Syndrome	HIV/AIDS
Parvovirus	Spina Bifida	Sudden Infant Death
#3) According to your knowledge how can CMV infection be acquired?		
Direct skin contact	Urine contact	Blood contact
Breastfeeding	Sexual intercourse	Transfusion/Transplant
Saliva contact	Air conduction	Do not know
#4) Which are the symptoms at birth in newborns with cCMV infection?		
No symptoms	Heart defect	Prematurity/Dismaturity
Harelip	Hearing loss	Microcephaly
Death	Convulsion	Spina bifida
Petechiae	Phocomelia	Club-foot
#5) Which is/are the long-term effects in children with cCMV infection?		
None of them	Blindness	Psycho-motor retardation Do not know
Hearing loss	Obesity	Death
#6) Which behaviors pregnant woman should assume to prevent cCMV infection?		
Avoid saliva-sharing		Taking a vitamin supplement
Not share toothbrush or other bath tools		Vaccine/medicine
Not share cutlery with children		Not have animal contact
Hand-wash after diaper change		Not eat rare cooked meat
If already immunised against CMV is useless to observe women’s behaviour		Not eat dairy products
Change the cats’ litter box with gloves		

The web-questionnaire was available on line from 14^th^ May to 1^st^ July 2015 and weekly e-mail reminders were sent to enhance participation. An informational brochure about CMV (the virus, its epidemiology and its behaviour) was downloadable for participants who completed the survey both as an incentive for their enrolment and to obtain awareness feedback. The only criterion for exclusion from the survey was non-completion of the whole interview.

### Data analysis

Statistical analysis was performed with SPSS 22 (IBM SPSS Statistics) and Stata 13 (StataCorp LP College station, TX, USA). Awareness of CMV and cCMV according to respondent characteristics was assessed by using univariate analysis. In addition, multivariate logistic regression was conducted. Odds ratios and 95 % confidence intervals were calculated.

Awareness among sub-populations was compared using Chi-square test for categorical variable and the level of significance was set at Sidak-adjusted *p*-values below 0.05.

An overall score calculated per questionnaire item, based on the difference between the sum of right answers minus the sum of wrong answers, assigning one point per correct answer and one point per wrong answer, was computed. The maximum and minimum achievable scores depend on the questionnaire item as reported in Table 6. For a better comparison in relation to the four key questions concerning knowledge of CMV infection, normalized mean scores (n.m.s.) were calculated using the following formula: (X-Xmin) / (Xmax-Xmin)*10. F’s exact test was used to compare mean scores of different groups and Sidak correction for multiple-comparison test. Differences were considered significant at a *p*-value <0.05.

## Results

The study includes 10,190 respondents out of 70,795 attendees at the University of Milan (14.4 %); 69.2 % were students, 21.4 % belonged to the academic staff and the 9.4 % to the non-academic staff. 65.7 % were female, the mean age was 30.2 years (std. dev. 11.9), 45.4 % were graduates (Bachelor or Master) and 83.7 % were non-parents (Table 2).

**Table 2:** Awareness of CMV (#1) and cCMV (#2) according to respondent characteristics

		Gender		Age			Education			Parenthood		Total
		Female	Male	<24 years	25–40 year	>40 year	Undergraduates	Bachelor graduates	Masters graduates	Non-parents	Parents	
Total respondents	n.	6,698	3,492	4,356	3,081	1,718	5,561	1,810	2,819	8,530	1,660	10,190
	%	65.7	34.3	47.6	33.7	18.8	54.6	17.8	27.7	83.7	16.3	100.0
#1 Responders heard of CMV	n.	3,609	1,742	1,604	1,849	1,246	2,491	771	2,089	4,008	1,343	5,351
	%	53.9	49.9	36.8	60.0	72.5	44.8	42.6	74.1	47.0	80.9	52.5
Univariate analysis	OR (95 % C.I)	1	0.85 (0.79–0.92)	1	2.57 (2.34–2.83)	4.53 (4.01–5.12)	1	0.91 (0.82–1.01)	3.53 (3.20–3.90)	1	4.7 (4.20–5.44)	
Multivariate analysis	OR (95 % C.I)	1	0.75 (0.68–0.83)	1	1.91 (1.71–2.15)	1.76 (1.43–2.159)	1	0.8 (0.71–0.90)	1.4 (1.14–1.72)	1	2.9 (2.43–3.47)	
#2 Responders heard of CMV and that it can be congenital	n.	2,255	961	842	1,187	812	1,392	363	1,461	2,261	955	3,216
	%	62.5	55.2	52.5	64.2	65.2	55.9	47.1	69.9	56.4	71.1	60.1
Univariate analysis	OR (95 % C.I)	1	0.74 (0.66–0.83)	1	1.6 (1.42–1.86)	1.6 (1.45–1.97)	1	0.7 (0.60–0.83)	1.84 (1.63–2.08)	1	1.9 (1.66–2.17)	
Multivariate analysis	OR (95 % C.I)	1	0.68 (0.60–0.77)	1	1.25 (1.06–1.48)	0.827 (0.64–1.06)	1	0.62 (0.52–0.74)	1.4 (1.09–1.81)	1	2.02 (1.67–2.45)	

Overall, 52.5 % (5,351/10,190) of respondents had already heard of CMV and with the exception of Fetal Alcohol Syndrome (41 %), CMV was the least known among the other congenital conditions, namely HIV/AIDS (99.9 %), Down Syndrome (99.8 %), Congenital Rubella Syndrome (97.8 %), Spina bifida (80.2 %), Congenital Toxoplasmosis (78,4 %), Sudden Infant Death Syndrome (63.6 %) and Parvovirus infection (56.4 %).

According to both univariate and multivariate analyses, having heard of CMV was significantly lower in men (49.9 % *vs* 53.9 %) and higher in parents (80.9 % *vs* 47.0 %) and significantly increased both with age (person <25 years *vs* 25–40 year *vs* >40 year: 36.8 % *vs* 60.0 % *vs* 72.5 %) and level of education (Masters graduates *vs* Undergraduates: 74.1 % *vs* 44.8 %) (Table 2).

Out of the 5,351 individuals who had already heard of CMV, 3,216 (60.1 %) realized that the virus is able to cause congenital infection. According to both statistical analyses, women (62.5 %), people aged more than 24 years (64.2 and 65.2 % in people clustered in 25–40 year and >40 year, respectively), Master-graduates (69.9 %) and parents (71.1 %) recognised cCMV most frequently (Table 2). The 31.5 % (3,216/10,190) of all the people enrolled in the study declared to know that CMV can be congenitally acquired.

People aware of cCMV were asked to continue the questionnaire in order to test the quality of their information about viral transmission routes, the outcomes of the infection in children and preventive measures.

Among these 3,216 individuals, about 16 % stated not to know how cCMV transmission occurs, while saliva (67.9 %), blood contact (67.6 %), sexual transmission (58.4 %) and transfusion/transplant (53.8 %) were the most recognised means of viral spread. Infection by urine and breastfeeding were the via least identified by 42.7 and 42.2 % of respondents respectively (Table 3). According to the scores achieved and statistical results, men (*F* = 5.0, *p*-value = 0.025), people aged from 25 to 40 year (*F* = 4.2, *p*-value = 0.015) and without children (*F* = 26.0, *p*-value = 0.000) were those who showed the greatest awareness of the infection transmission routes (Table 6).

**Table 3:** Knowledge concerning the route of transmission of CMV (#3)

		Gender (*n* = 3,215)		Age (*n* = 2,840) ^a^			Education (*n* = 3,215)			Parenthood (*n* = 3,215)		Total
		Female	Male	<24 years	25–40 year	>40 year	Undergraduates	Bachelor graduates	Masters graduates	Non-parents	Parents	
Total of respondents (n.)			961	842	1,186	812	1,391	363	1,461	2,260	955	3,215
Right answers (%)	Saliva	67.0	70.1	65.7	71.9	68.1	66.4	65.0	70.1	67.6	68.6	67.9
	Sexual Intercourse	56.4*	63.2	61.2	56.8	58.7	60.0	59.8	56.6	60.4*	53.7	58.4
	Blood contact	65.9*	71,4	69.1	67.5	66.1	68.6	71.4	65.6	69.6*	62.8	67.6
	Urine contact	43.2	41.5	41.6	42.6	44.6	42.0	43.8	43.1	42.6	42.9	42.7
	Breast-feeding	42.7	41.1	50.4*	40.7	33.4	45.5*	41.9	39.2	47.0*	30.8	42.2
	Transfusion/Transplant	51.6*	59.1	58.7*	56.4	46.6	54.7	54.3	52.9	58.2*	43.6	53.8
Wrong answers (%)	Direct skin contact	4.6	4.8	3.9	4.6	6.0	4.3	2.5	5.5	4.4	5.1	4.6
	Air	9.9	7.8	8.3	10.5	9.5	9.3	5.2	10.3	8.2*	11.7	9.3
	Do not know	15.6	15.9	15.7	13.9	16.8	16.0	14.3	15.8	15.4	16.4	15.7

^a^ Information about the age of responders was provided by 2,840 out of 3,215 individuals

* *p* < 0.05

Among others results, hearing loss was recognised as a neonatal symptom of cCMV manifest at birth or in infancy by 54.8 and 56.2 % respectively while 46 % of individuals believed that ‘No symptoms of cCMV infection may be shown at birth’ and 29.8 % declared that they did not know the long-term effects caused by cCMV (Table 4). Awareness of both neonatal symptoms and long-term effects of cCMV infection was significantly higher in the absence of parenthood (#4: *F* = 17.1, *p*-value = 0.000. #5: F =17.3, *p*-value = 0.000) and less recognised by older people (#4: *F* = 19.0, *p*-value =0.000. #5: *F* = 15.4, *p*-value = 0.000). Undergraduates showed a better recognition of cCMV-sequelae than others did (*F* = 4.3, *p*-value =0.014), while no significant difference was recorded when gender was inspected (Table 6).

**Table 4:** Knowledge concerning symptoms at birth (#4) and long-terms effects (#5) of cCMV infection

		Gender (*n* = 3,215)		Age (*n* = 2,840) ^a^			Education (*n* = 3,215)			Parenthood (*n* = 3,215)		Total
		Female	Male	<24 years	25–40 year	>40 year	Undergraduates	Bachelor graduates	Masters graduates	Non-parents	Parents	
Total of respondents (n.)		2,254	961	842	1,186	812	1,391	363	1,461	2,260	955	3,215
Symptoms of cCMV infection showed at birth (#4)												
Right answers (%)	No symptoms	44.9	48.6	43.1	49.4	47.0	44.9	42.4	48.1	45.8	46.7	46.0
	Petechiae	35.7	37.3	37.9*	39.1	30.1	38.3	34.4	34.6	39.7*	27.6	36.1
	Prematurity/SGA	52.3	46.8	53.6*	55.2	42.9	51.8	49.9	49.8	52.6*	56.2	50.7
	Hearing Loss	56.3	51.3	56.1	61.1	46.1	55.8	53.2	54.4	56.4	51.2	54.8
	Convulsions	36.6	35.5	34.0*	41.6	30.2	37.3	33.3	35.9	39.0*	29.7	36.2
	Microcephaly	44.7	43.4	47.3*	50.8	32.8	46.3	41.3	43.1	47.9*	35.8	44.3
	Death	36.5	38.8	42.9*	41.7	25.1	40.9*	31.1	35.2	41.2*	27.6	37.2
Wrong answers (%)	Spina Bifida	6.7	9.5	10.6*	7.1	4.8	10.2*	8.0	4.9	8.6*	5.1	7.6
	Club Foot	4.0	4.1	5.7*	3.9	2.5	5.7*	2.8	2.7	4.5	2.8	4.0
	Harelip	6.1	4.9	9.1*	5.6	2.8	8.3*	6.3	3.1	6.7*	3.4	5.7
	Phocomelia	8.5	7.4	11.6	8.7	4.3	9.6	9.6	6.5	9.3*	5.5	8.2
	Cardiac defect	34.3	31.5	40.4*	37.9	22.4	37.0*	30.3	30.8	36.5*	26.2	33.4
Long-terms effects of cCMV infection (#5)												
Right answers (%)	Psychomotor retardation	53.7	53.7	54.8*	58.9	47.5	55.6	54.8	51.5	55.7*	49.0	53.7
	Death	14.5	17.7	16.4	14.8	15.3	17.1	15.4	13.8	16.5	12.9	15.4
	Blindness	43.7	41.2	45.1*	47.0	39.0	44.4	40.8	42.2	44.4	39.7	43.0
	Hearing Loss	57.8*	52.5	58.8*	61.8	48.3	58.2	55.4	54.4	58.3*	51.1	56.2
Wrong answers (%)	Obesity	0.8	0.7	0.7	0.8	0.4	0,9	1.4	0.6	0.9	0.4	0.8
	None of them	1.2	1.8	1.9	1.1	1.0	1.6	1.1	1.2	1.5	1.2	1.4
	Don’t know	29.2	31.2	24.7*	26.3	37.4	26.5*	30.6	32.9	27.7*	34.8	29.8

^a^ Information about the age of responders was provided by 2,840 out of 3,215 individuals. * *p* < 0.05

About 72 and 75 % of interviewees considered ‘not sharing a toothbrush and other bathroom utensils’ and ‘avoid sharing saliva’ respectively, as appropriate behaviours during pregnancy. ‘Do not share cutlery with children’ and ‘to hand-wash after diaper changes’ were recognised by 57 and 55.6 % of individuals, respectively. More than a third of people believed that ‘If already immunised against CMV women’s behaviour is irrelevant’ while ‘change the cat litter box with gloves’, ‘do not eat rare cooked meat’ and ‘do not have animal contact’ were considered as a preventive measures against cCMV by 35.4 %, 26.7 %, and 28.5 % respectively (Table 5). Individuals aged more than 24 (*F* =20.2, *p*-value = 0.000), Masters graduates (*F* = 14.3, *p*-value = 0.000) and parents (*F* = 17.8, *p*-value = 0.000), were significantly better informed on cCMV prevention while gender did not have any influence on awareness (Table 6).

**Table 5:** Knowledge concerning pregnant women’s behavior to prevent cCMV infection (#6)

		Gender (*n* = 3,215)		Age (*n* = 2,840) ^a^			Education (*n* = 3,215)			Parenthood (*n* = 3,215)		Total
		Female	Male	<24 years	25–40 year	>40 year	Undergraduates	Bachelor graduates	Masters graduates	Non-parents	Parents	
Total of respondents (n.)		2,254	961	842	1,186	812	1,391	363	1,461	2,260	955	3,215
Right answers (%)	Avoid saliva-sharing	72.1	70.7	70.1	74.5	73.5	71.0	67.5	73.4	70.2	75.1	71.7
	Not share toothbrush or other bath tools	75.6	73.5	75.7	77.3	75.6	75.0	73.0	75.4	74.6	75.9	75.0
	Not share cutlery with children	57.9	54.9	54.2*	62.1	56.3	55.5	53.4	59.4	56.0	59.6	57.0
	Hand-wash after diaper change	57.3*	51.4	53.1	55.7	59.4	55.6	57.3	55.0	54.1	59.1	55.6
Wrong answers (%)	Taking a vitamin supplement	12.2	12.8	19.2*	10.5	8.6	15.9*	16.8	7.9	14.5*	7.3	12.3
	Vaccine/medicine	17.1*	23.3	28.9*	16.2	13.9	26.2*	19.3	12.1	22.0*	11.9	19.0
	Not have animal contact	28.7	28.1	35.2*	26.1	26.1	30.3*	34.7	25.2	30.0*	24.8	28.5
	Not eat rare cooked meat	27.6	24.9	34.2*	24.0	23.8	28.3*	36.9	22.7	28.1	23.7	26.7
	Not eat dairy products	7.1	6.4	8.3	6.8	5.3	7.3	8.5	6.1	7.7	5.1	6.9
	Change the cats’ litter box with gloves	36.9	31.7	42.5*	31.6	35.0	38.3*	44.9	30.3	36.8	31.9	35.4
	If already immunised against CMV is useless to observe women’s behavior	34.9	29.9	22.7*	39.2	37.9	27.3*	23.1	41.8	28.7*	44.6	33.4

^a^ Information about the age of responders was provided by 2,840 out of 3,215 individuals. * *p* < 0.05

**Table 6:** Mean Score (ms) and normalized Mean Score (n.ms) about knowledge of cCMV infection

		Gender (*n* = 3,215)		Age (*n* = 2,840) ^a^			Education (*n* = 3,215)			Parenthood (*n* = 3,215)		Total
		Female	Male	<24 years	25–40 year	>40 year	Undergraduates	Bachelor graduates	Masters graduates	Non-parents	Parents	ms
Route of transmission	ms [min = −3 max = 6]	3.0	3.2	3.2	3.1	2.9	3.1	3.1	3.0	3.2	2.7	3
	n.ms [0–10]	6.7	6.9	6.9	6.8	6.6	6.8	6.8	6.7	6.9	6.3	6.7
	F (*p*-value) and Sidak m-c test	5.0 (0.025) (a)		4.2 (0.015) (b)			1.2 (0.301)			26.0 (0.000) (a)		
Symptoms showed at birth	ms [min = −5 max = 7]	2.5	2.4	2.4	2.8	2.2	2.4	2.3	2.5	2,6	2,2	2.5
	n.ms [0–10]	6.3	6.2	6.2	6.5	6.0	6.2	6.1	6.3	6,3	6.0	6.3
	F (*p*-value) and Sidak m-c test	0.14 (0.707)		19.0 (0.000) (a) (c)			1.9 (0.148)			17,1 (0,000) (a)		
Long-term effects	ms [min = −3 max = 4]	1.4	1.3	1.5	1.5	1.1	1.5	1.3	1.3	1.4	1.2	1.4
	n.ms [0–10]	6.3	6.1	6.4	6.4	5.9	6.4	6.1	6.1	6.3	6.0	6.3
	F (*p*-value) and Sidak m-c test	1.1 (0.299)		15.4 (0.000) (b) (c)			4.3 (0.014) (b)			17.3 (0.000) (a)		
Pregnant women’s behaviours to prevent CMV infection	ms [min = −7 max = 4]	1.0	0.9	0.6	1.2	1.1	0.8	0.7	1.2	0.9	1.2	1
	n.ms [0–10]	7.3	7.2	6.9	7.5	7.4	7.1	7.0	7.5	7.2	7.5	7.3
	F (*p*-value) and Sidak m-c test	0.4 (0.522)		20.2 (0.000) (a) (b)			14.3 (0,000) (b) (c)			17.8 (0.000) (a)		
Total	ms	2.0	2.0	1.9	2.2	1.8	2.0	1.9	2.0	2.0	1.8	
	F (*p*-value) and Sidak m-c test	0.1 (0.812)		11.1 (0.000) (a) (c)			1.0 (0.386)			10.33 (0.001) (a)		

(a) Statistically significant difference between item 1 and item 2 base on Sidak multiple-comparision test (Sidak m-c test)

(b) Statistically significant difference between item 1 and item 3 base on Sidak multiple-comparision test

(c) Statistically significant difference between item 2 and item 3 base on Sidak multiple-comparision test

^a^ Information about the age of responders was provided by 2,840 out of 3,215 individuals

According to the overall results, the best-known aspects of cCMV concerned transmission routes (n.ms = 6.3) and pregnancy behaviours (n.m.s. = 7.3) and the most informed categories were people aged 25–40 years (*F* = 11.1, *p*-value = 0,000) and those without children (*F* = 10.33, *p*-value = 0,000).

## Discussion and conclusions

Our survey collected information regarding awareness of cCMV in a sizeable population with considerable heterogeneity of socio-demographic variables through a web-questionnaire. In Italy, as is the case in other countries, no data about cCMV awareness in general populations are available as yet. To our knowledge, the surveys published so far about this issue targeted specific population clusters such as pregnant women [6, 7], physicians [13], health care providers [9] and medical students [10].

In our study only about one third of the population inspected was aware that CMV can be congenitally transmitted although half of the participants had declared that they had heard of the virus and the factors associated with awareness of cCMV were womanhood, parenthood, people >24 years and Master’s degree holders.

Nevertheless, our population evinced hearing about cCMV more frequently than the one involved in a study carried out by Cannon et al., since in that study among 4,184 interviewees only the 10 % (13 % of women and the 7 % of men) stated a knowledge of cCMV [12]. This difference can be explained thanks to the high level of education of our respondents, given that almost all had, at least, achieved high school graduation. Moreover, cCMV prenatal screening has been relatively common in Italy whereas it is rare in the U.S.

As in others studies [6, 7, 14–16], also in our research awareness of cCMV ranked second lowest (fetal alcohol syndrome is the least recognised) among a group of congenitally acquired conditions all of which have an incidence in the population lower than that of cCMV. A number of reasons could lie behind this finding: first, there is no CMV vaccine, in contrast to other preventable infections such as rubella and measles; second, cCMV infection is less discussed in the media than toxoplasmosis and HIV/AIDS; third, children with cCMV do not have characteristics symptoms and signs such as those found in Down syndrome or spina bifida and so are typically not diagnosed. Since only 1 baby in every 10 infected shows symptoms at birth [1], cCMV infection may have low emotional impact on the population.

In spite of cCMV being the most important infectious cause of paediatric deafness [17], only around half of our interviewees recognised sensorineural hearing loss as a sequela (at birth or as a later development) of the congenital infection, similar to another study [8, 12], and they believed that cCMV infection is symptomatic at birth. The low quality of knowledge in the whole population concerning viral outcomes confirms that although people had already heard of cCMV, the virus was considered to be of little danger and potential damage, similar to Willame et al. results [16].

In our survey, the questions regarding both the transmission routes and the suggested preventive behaviours computed the highest scores, suggesting a generally good health education that, however, is not specific for cCMV. In fact, when each answer is considered in detail, some findings have to be underlined. First, although more than half of individuals reported knowing that the infection can result from adult-to-adult transmission (such as via blood, sexual activity, transfusion/transplant), they were much less aware that children –in particular through urine-are involved in CMV infection, similar to others studies [6, 11, 12]. So, it can be assumed that our population failed to recognise CMV shedding from infected children, both congenitally and postnatally, and to realize that child-to-child and child-to-mother are the most common routes of viral transmission [18], especially when the child attends a day-care centre [19]. Consequently, among preventive measures that pregnant women should follow in order to avoid the infection, behaviours specifically concerning child contact were recognised by up to twenty percentage points lower than others items. Second, about a third of respondents thought that ‘avoiding eating rare cooked meat’, ‘changing the cat litter box with gloves’ and ‘avoiding animal contact’ were preventive behaviours against cCMV, mistaking CMV for Toxoplasma. Third, women, individuals of childbearing age and parents, believed, in percentages ranging from 35 to 45 %, that pregnant women are not at risk of infection if already immunized, thus providing further evidence that people do not know that CMV behaves differently from “conventional” infectious agents. In fact, the presence of CMV antibodies in the serum is often wrongly referred to as “immunity” (immunity being interpreted as protective). However, reinfection with CMV can occur in CMV seropositive people because the protection conferred by preconception immunity is limited to strains-dependent immune responses [20] and viral reactivation can take place since CMV can be latent in the host after primary infection [21].

Unexpectedly, women and parents did not show an increased degree of knowledge about cCMV in our study, probably due to being poorly informed. A number of studies have shown that physicians, obstetricians and health care providers are not familiar with cCMV transmission, prevention and management even if they are the main source of child and maternal health information [8, 9, 22]. In an earlier Italian survey, the proportion of puerpera declaring themselves to have received information about appropriate behaviours against cCMV was about 26 % in the ‘immune’ group *vs* the 49 % in the ‘non-immune’ (*p* < 0.000) (immune here referring to their CMV serological status) (Barbi M., et al., *unpublished data*). Considering an ‘immune’ woman safe from further CMV infections means giving her a false sense of security.

Moreover, some authors have reported a paucity of CMV information in pregnancy-related reference books and websites [23, 24]. This aspect should be investigated in more depth in future Italian studies.

The most complete degree of awareness about cCMV was linked to childbearing-age and, surprisingly, with not having children, even if non-parent results showed less of them having heard of cCMV when compared to parents. We regressed the “knowledge of route of transmission mean scores” on parenthood controlling for age, education and gender. The regression analysis shows that this finding does not depend on the different composition of the individuals clustered in the parents *vs* ‘non-parents’ group. In any case, many respondents who had heard of cCMV had had their youngest child several years before so they may have forgotten the information given about the virus, if indeed it had ever been received (data not shown). Additional investigation is needed to explore this finding.

The study was subject to several shortcomings. First, the response rate was 14.4 %. Despite this low rate, it represents more than 10,000 interviewees, hence, as far as we are aware, this is the largest survey ever carried out about cCMV awareness. Second, the survey was based on a convenience sample that, although used widely by researchers, can lead to biased results. Interviewees had an higher education level, were younger on average, and the gender distribution was skewed towards female in comparison to the general Italian population. In any case, this study does not focus on any particular group but on a population that is heterogeneous for gender, age, level of education and parenthood. Third, differences between respondents and non-respondents could have resulted in biased results. Even if we had no information on non-respondents, the declaration of a public health issue as the general topic in the email invitation leads us to suppose that respondents answered because they may have been more interested in health issues than non-respondents. This bias, therefore, might imply that the true awareness concerning cCMV is less than that reported above.

In conclusion our study highlights the poor, confused and insufficient quality and degree of awareness about cCMV in the considered population although it was conducted among individuals predominantly possessing a high level of education and being of childbearing age. As a consequence of our findings, we strongly believe that various actions have to be taken by public health policy-makers and these findings could be useful for determining both the most effective content for preventive messages and the most appropriate target populations; these actions include the following: all employees involved in child-mother care have to be adequately educated about cCMV so as to be able to share accurate information both on cCMV outcomes and on appropriate preventive behaviour. Health education programmes targeting cCMV should urgently address and remedy the problems revealed here and a media campaign should be launched to raise awareness.

## Abbreviations

CCMV: Congenital cytomegalovirus
CMV: Cytomegalovirus
n.m.s.: Normalized mean scores

## References

[1] Kenneson A, Cannon MJ (2007). Review and meta-analysis of the epidemiology of congenital cytomegalovirus (CMV) infection. Rev Med Virol.

[2] Bello C, Whittle H (1991). Cytomegalovirus infection in Gambian mothers and their babies. J Clin Pathol.

[3] Zhang XW, Li F, Yu XW, Shi XW, Shi J, Zhang JP (2007). Physical and intellectual development in children with asymptomatic congenital cytomegalovirus infection: a longitudinal cohort study in Qinba mountain area, China. J Clin Virol.

[4] Cannon MJ (2009). Congenital cytomegalovirus (CMV) epidemiology and awareness. J Clin Virol.

[5] Vauloup-Fellous C, Picone O, Cordier AG, Parent-du-Châtelet I, Senat MV, Frydman R (2009). Does hygiene counseling have an impact on the rate of CMV primary infection during pregnancy? Results of a 3-year prospective study in a French hospital. J Clin Virol.

[6] Lim SL, Tan WC, Tan LK (2012). Awareness of and attitudes toward congenital cytomegalovirus infection among pregnant women in Singapore. Int J Gynaecol Obstet.

[7] Cordier AG, Guitton S, Vauloup-Fellous C, Grangeot-Keros L, Ayoubi JM, Benachi A (2012). Awareness of cytomegalovirus infection among pregnant women in France. J Clin Virol.

[8] Korver AM, De Vries JJ, De Jong JW, Dekker FW, Vossen AC, Oudesluys-Murphy AM (2009). Awareness of congenital cytomegalovirus among doctors in the Netherlands. J Clin Virol.

[9] Cordier AG, Guitton S, Vauloup-Fellous C, Grangeot-Keros L, Benachi A, Picone O (2012). Awareness and knowledge of congenital cytomegalovirus infection among health care providers in France. J Clin Virol.

[10] Baer HR, McBride HE, Caviness AC, Demmler-Harrison GJ (2014). Survey of congenital cytomegalovirus (cCMV) knowledge among medical students. J Clin Virol.

[11] Jeon J, Victor M, Adler SP, Arwady A, Demmler G, Fowler K (2006). Knowledge and awareness of congenital cytomegalovirus among women. Infect Dis Obstet Gynecol.

[12] Cannon MJ, Westbrook K, Levis D, Schleiss MR, Thackeray R, Pass RF (2012). Awareness of and behaviors related to child-to-mother transmission of cytomegalovirus. Prev Med.

[13] Ross DS, Rasmussen SA, Cannon MJ, Anderson B, Kilker K, Tumpey A (2009). Obstetrician/gynecologists’ knowledge, attitudes, and practices regarding prevention of infections in pregnancy. J Womens Health (Larchmt).

[14] Ross DS, Victor M, Sumartojo E, Cannon MJ (2008). Women’s knowledge of congenital cytomegalovirus: results from the 2005 HealthStyles survey. J Womens Health (Larchmt).

[15] Pereboom MT, Manniën J, Spelten ER, Schellevis FG, Hutton EK (2013). Observational study to assess pregnant women’s knowledge and behaviour to prevent toxoplasmosis, listeriosis and cytomegalovirus. BMC Pregnancy Childbirth.

[16] Willame A, Blanchard-Rohner G, Combescure C, Irion O, Posfay-Barbe K, Martinez De Tejada B (2015). Awareness of Cytomegalovirus Infection among Pregnant Women in Geneva, Switzerland: A Cross-sectional Study. Int J Environ Res Public Health.

[17] Barbi M, Binda S, Caroppo S, Ambrosetti U, Corbetta C, Sergi P (2003). A wider role for congenital cytomegalovirus infection in sensorineural hearing loss. Pediatr Infect Dis J.

[18] Cannon MJ, Hyde TB, Schmid DS (2011). Review of cytomegalovirus shedding in bodily fluids and relevance to congenital cytomegalovirus infection. Rev Med Virol.

[19] Bale JF, Zimmerman B, Dawson JD, Souza IE, Petheram SJ, Murph JR (1999). Cytomegalovirus transmission in child care homes. Arch Pediatr Adolesc Med.

[20] Boppana SB, Rivera LB, Fowler KB, Mach M, Britt WJ (2001). Intrauterine transmission of cytomegalovirus to infants of women with preconceptional immunity. N Engl J Med.

[21] Larsson S, Söderberg-Nauclér C, Möller E (1998). Productive cytomegalovirus (CMV) infection exclusively in CD13-positive peripheral blood mononuclear cells from CMV-infected individuals: implications for prevention of CMV transmission. Transplantation.

[22] (CDC) CfDCaP (2008). Knowledge and practices of obstetricians and gynecologists regarding cytomegalovirus infection during pregnancy--United States, 2007. MMWR Morb Mortal Wkly Rep.

[23] Thackeray R, Wright A, Chipman K (2014). Congenital cytomegalovirus reference material: a content analysis of coverage and accuracy. Matern Child Health J.

[24] Read JS, Cannon MJ, Stanberry LR, Schuval S (2008). Prevention of mother-to-child transmission of viral infections. Curr Probl Pediatr Adolesc Health Care.

